# Inhibition of ferroptosis promotes megakaryocyte differentiation and platelet production

**DOI:** 10.1111/jcmm.17289

**Published:** 2022-05-13

**Authors:** Baoquan Song, Wenjing Miao, Qingya Cui, Bingyu Shi, Jian Zhang, Huiying Qiu, Leisheng Zhang, Yue Han

**Affiliations:** ^1^ National Clinical Research Center for Hematologic Diseases Jiangsu Institute of Hematology The First Affiliated Hospital of Soochow University Suzhou China; ^2^ Institute of Blood and Marrow Transplantation Soochow University Suzhou China; ^3^ Key Laboratory of Thrombosis and Hemostasis of Ministry of Health Suzhou China; ^4^ The Postdoctoral Research Station College of Life Science Nankai University Tianjin China; ^5^ Precision Medicine Division Health‐Biotech (Tianjin) Stem Cell Research Institute Co., Ltd. Tianjin China

## CONFLICT OF INTEREST

The authors declared that they have no conflict of interest.

## AUTHOR CONTRIBUTIONS

**Baoquan Song:** Data curation (equal); Funding acquisition (equal); Methodology (equal); Project administration (equal); Validation (equal); Writing – original draft (equal); Writing – review & editing (equal). **Wenjing Miao:** Data curation (equal); Methodology (equal); Project administration (equal); Writing – original draft (equal); Writing – review & editing (equal). **Qingya Cui:** Data curation (equal); Investigation (equal); Methodology (equal); Project administration (equal); Writing – original draft (equal); Writing – review & editing (equal). **Bingyu Shi:** Data curation (equal); Methodology (equal); Project administration (equal). **Jian Zhang:** Data curation (equal); Funding acquisition (equal). **Huiying Qiu:** Data curation (equal); Funding acquisition (equal). **Leisheng Zhang:** Conceptualization (equal); Writing – review & editing (equal). **Yue Han:** Conceptualization (equal); Writing – review & editing (equal).

## CONSENT STATEMENT

Informed consent was obtained from all individual participants included in the study.

Iron overload (IO), as a consequence of chronic blood transfusions, ineffective haematopoiesis or abnormal regulation of hepcidin, is a common condition in patients with haematological malignancies and haematopoietic stem cell transplantation (HSCT) recipients.^1^ Researches have shown that iron overload can damage bone marrow microenvironment and vital organs, injuring the haematopoiesis.^2^, ^3^ Ferroptosis, an iron‐dependent nonapoptotic cell death, has been identified in cancer cells, tissue injury and T‐cell immunity.^4^, ^5^, ^6^ Recently, it has been shown that ferroptosis regulated several fundamental cellular processes including cell proliferation and differentiation.^7^, ^8^ Herein, we explored the potential role of ferroptosis in the impaired haematopoiesis caused by IO.

**FIGURE 1:**
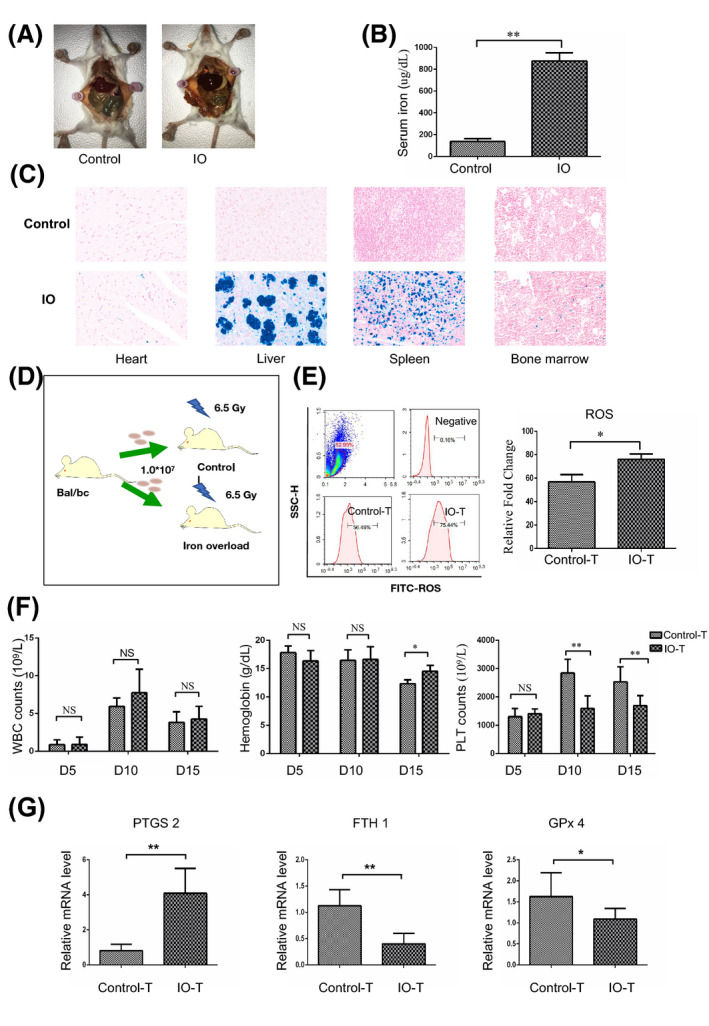
Ferroptosis contributed to megakaryopoiesis dysfunction in IO recipients. (A) Macroscopic findings of iron‐overloaded mice, intraperitoneally injected with 50 mg of iron‐dextran in total. Control mice were injected with phosphate‐buffered saline. (B) Serum iron concentration after 4 weeks of injection. (C) Histological examinations (iron stain) of vital organs. In IO mice, depositions of excess iron (stained in blue) are distributed in the heart, liver, spleen and bone marrow (×200). (D) Schematic diagram of bone marrow transplantation (BMT). (E) Flow cytometric analysis of reactive oxidative species (FITC‐ROS) in bone marrow mononuclear cells (MNCs). (F) WBC (white blood cells), Hb (haemoglobin) and PLT (platelet) in the peripheral blood of control and IO recipients after BMT. (G) qRT‐PCR detecting ferroptosis related genes expression, including PTGS2, FTH1and GPx4. Values are mean SD calculated in 8 control and 8 iron‐overloaded mice *p < 0.05 and **p < 0.01

Iron overload mouse model was established by intraperitoneally injecting with iron dextran (25 mg/ml) every 3 days for 4 weeks according to the previous study. IO mice showed significantly higher serum iron concentration and excess iron accumulation in vital organs including heart, liver, spleen and bone marrow when compared with control group (**p < 0.01; Figure 1A‐C). Peripheral blood counts of IO mice were not remarkably affected (*p* > 0.05; Figure S1A). However, IO recipients that received bone marrow transplantation (BMT) from normal donors showed delayed platelet reconstruction, while the number of white blood cells (WBCs) did not show significant difference between IO and normal recipients. Compared with normal recipients, there is a slight increase in haemoglobin (Hb) level in IO recipients. (*p < 0.05, **p < 0.01; Figure 1D,F). These findings indicated that excess iron affect platelet recovery in IO mice after bone marrow transplantation.

Ferroptosis is a nonapoptotic cell death that can be activated by excess iron. A low iron environment promotes megakaryocyte (MK) bias of megakaryocytic‐erythroid progenitors (MEP) and low iron context favours platelet synthesis.^9^, ^10^ Therefore, in order to explore whether excess iron cause delayed platelet reconstruction by inducing ferroptosis, we detected reactive oxygen species (ROS) level, which plays critical role in ferroptosis, in bone marrow mononuclear cells (BM MNCs). We found that there was a significant increase in ROS level in the IO recipients (*p < 0.05; Figure 1E). Ferroptosis regulation‐related genes, such as FTH1, GPx4 and PTGS2, were also assessed by quantitative RT‐PCR (qRT‐PCR). The primers used for qRT‐PCR are listed in Table 1. The expression of FTH1 and GPx4 in the IO recipients was decreased while the expression of PTGS2 was increased when compared with normal recipients (*p < 0.05, **p < 0.01; Figure 1G). These findings indicated that ferroptosis may involve in the delayed platelet reconstruction caused by IO.

**TABLE 1:** Primer sequences of genes evaluated by qRT‐PCR

Gene	Primer (forward) 5′−3′	Primer (Reverse) 5′−3′
PTGs2 (Human)	CTGCGCCTTTTCAAGGATGG	GGGGATACACCTCTCCACCA
FTH1 (Human)	TCCTACGTTTACCTGTCCATG	CTGCAGCTTCATCAGTTTCTC
GPx4 (Human)	GCAACCAGTTTGGGAGGCAGGAG	CCTCCATGGGACCATAGCGCTTC
PTGs2 (mouse)	CTGCGCCTTTTCAAGGATGG	GGGGATACACCTCTCCACCA
FTH1 (mouse)	GCCGAGAAACTGATGAAGCTGC	GCACACTCCATTGCATTCAGCC
GPx4 (mouse)	CCTCCCCAGTACTGCAACAG	GGCTGAGAATTCGTGCATGG

**FIGURE 2:**
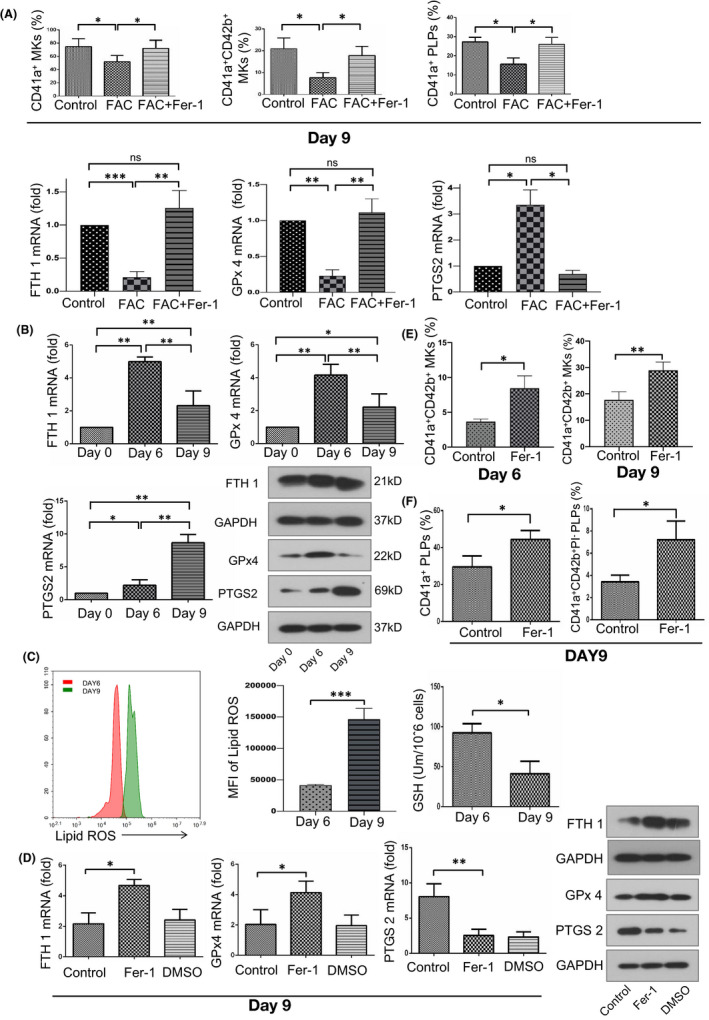
Suppressed ferroptosis promoted MK differentiation and platelet production. (A) Excessive iron impaired megakaryoctopoiesis by inducing ferroptosis. The percentage of CD41a+ and CD41a+CD42b+ MKs after treated with or without ferric ammonium citrate (FAC, 200 μM) for 9 days. The effect of FAC on expression of ferroptotic markers. (B) qRT‐PCR and Western‐blot analysis of expression of ferroptosis related factors (FTH1, GPx4 and PTGS2) during MK differentiation. (C) The reduction of GSH level and elevated lipid ROS level during MK differentiation. (D) Fer‐1 suppressed ferroptosis at the late stage of MK differentiation (day 9). (E) Flow cytometer analysis for percentage of CD41a+ CD42b+ MKs after Fer‐1 treatment on Day 6 and Day 9 of differentiation. (F) Percentage change of CD41a+, CD41a+CD42b+PI‐ PLPs in control or Fer‐1 groups, respectively. Data shown as mean ± SD. *p < 0.05 and **p < 0.01

In order to study the effect of excess iron on megakaryocytopoiesis, we established an in vitro iron overload culture system. Peripheral blood derived CD34^+^ cells (haematopoietic stem cells, HSCs) were incubated in serum free medium supplied with TPO, SCF and IL‐3 for 9 days. Megakaryocyte specific integrin CD41a and CD42b gradually appear in the process of megakaryocyte differentiation (FigureS 1B). We found that in the presence of 200 μM ferric ammonium citrate (FAC), the percentage of CD41a^+^ MKs and CD41a^+^CD42b^+^ MKs (represent mature MKs), was significantly decreased when compared with control group (Figure 2A). In addition, FAC also decreased CD41a^+^ platelet‐like particles (PLPs) production while CD41a^+^CD42b^+^PI^−^ PLPs could hardly be detected in FAC group (Figure 2A). In order to explore whether excessive iron impaired megakaryocytopoiesis by inducing ferroptosis, we analysed the expression of GPx4, FTH1 and PTGS2. GPx4 and FTH1 mRNA levels were dramatically decreased in FAC treated MKs while PTGS2 mRNA level was increased (Figure 2A). To further support the notion that excess iron could induce ferroptosis in MKs, ferrostatin‐1 (Fer‐1), a specific inhibitor of ferroptosis, was added to the medium, and we found that the above changes could be reversed by Fer‐1. Therefore, we think that excess iron impaired megakaryocytopoiesis by inducing ferroptosis.

To further explore whether ferroptosis participate in megakaryocyte differentiation, we investigated the concentration of glutathione (GSH) during the process of differentiation of CD34^+^ cells into MKs. We found that at the late stage of differentiation (Day 9), the concentration of GSH was significantly lower than that in the early stage of differentiation (Day 6; Figure 2C). Accumulation of lipid‐based reactive oxygen species (ROS) is an important feature of ferroptosis. Lipid ROS was measured with C11‐BODIPY and detected by flow cytometry. We observed enhanced lipid ROS levels in MKs on Day 9 than on Day 6 of differentiation (Figure 2C). In addition, we investigated in vitro mRNA expression of FTH1, GPx4 and PTGS2 during MK differentiation. The expression of PTGS2 was markedly elevated, and the level of FTH1 and GPx4 mRNA was decreased on Day 9 of differentiation compared with Day 6(*p < 0.05, **p < 0.01; Figure 2B). The alteration of FTH1, GPx4 and PTGS2 expression was further confirmed by Western blots (Figure 2B).

Furthermore, we explored whether Ferrostatin‐1 (Fer‐1), a specific inhibitor of ferroptosis could increase MK differentiation and platelet production. CD 34^+^ cells were induced into MK at the optimal concentration of Fer‐1 (10 μM) and cells were detected on 6 and 9 days of culture (Figure S2A). Fer‐1 treatment increased GPx4 and FTH1 gene expression and decreased PTGS2 expression. As well, GPx4 and FTH1 protein expression was significantly elevated while PTGS2 protein expression was reduced after incubation with Fer‐1 for 9 days (Figure 2D). In the Fer‐1 treated group, the percentage of CD41a^+^ MKs and CD41a^+^CD42b^+^ MKs was significantly increased when compared with the control group on Day 6 and Day 9 of culture (**p < 0.05; Figure 2E, Figure S2B). Meanwhile, we observed that more PLPs (CD41a^+^ or CD41a^+^CD42b^+^PI^−^) were generated from Fer‐1‐treated groups (*p < 0.05, **p < 0.01; Figure 2F). However, there was no significant influence on polyploidization of MKs (Figure S2C). These findings indicated that in vitro megakaryocyte differentiation process may be accompanied by the activation of ferroptosis and Fer‐1 could significantly enhanced MK differentiation and platelet production.

In summary, this study demonstrated that ferroptosis may participate in the process of megakaryopoiesis, and the inhibition of ferroptosis may be an alternative approach to counteract megakaryopoiesis dysfunction caused by IO.

## Supporting information

Fig S1

Fig S2

Supplementary Material

## References

[1] Koreth J , Antin JH . Iron overload in hematologic malignancies and outcome of allogeneic hematopoietic stem cell transplantation. Haematologica. 2010;95(3):364‐366. 20207843 10.3324/haematol.2009.017244 PMC2833064

[2] Zhang Y , Zhai W , Zhao M , et al. Effects of iron overload on the bone marrow microenvironment in mice. PLoS One. 2015;10(3):e0120219. 25774923 10.1371/journal.pone.0120219 PMC4361683

[3] Okabe H , Suzuki T , Uehara E , Ueda M , Nagai T , Ozawa K . The bone marrow hematopoietic microenvironment is impaired in iron‐overloaded mice. Eur J Haematol. 2014;93(2):118‐128. 24628561 10.1111/ejh.12309

[4] Dixon SJ , Lemberg KM , Lamprecht MR , et al. Ferroptosis: an iron‐dependent form of nonapoptotic cell death. Cell. 2012;149(5):1060‐1072. 22632970 10.1016/j.cell.2012.03.042 PMC3367386

[5] Mou Y , Wang J , Wu J , et al. Ferroptosis, a new form of cell death: opportunities and challenges in cancer. J Hematol Oncol. 2019;12(1):34. 30925886 10.1186/s13045-019-0720-y PMC6441206

[6] Li Q , Han X , Lan X , et al. Inhibition of neuronal ferroptosis protects hemorrhagic brain. JCI Insight. 2017;2(7):e90777. 28405617 10.1172/jci.insight.90777 PMC5374066

[7] Wang D , Xie N , Gao W , Kang R , Tang D . The ferroptosis inducer erastin promotes proliferation and differentiation in human peripheral blood mononuclear cells. Biochem Biophys Res Commun. 2018;503(3):1689‐1695. 30049441 10.1016/j.bbrc.2018.07.100 PMC6179365

[8] Gong Y , Wang N , Liu N , Dong H . Lipid peroxidation and GPX4 inhibition are common causes for myofibroblast differentiation and ferroptosis. DNA Cell Biol. 2019;38(7):725‐733. 31140862 10.1089/dna.2018.4541

[9] Xavier‐Ferrucio J , Scanlon V , Li X , et al. Low iron promotes megakaryocytic commitment of megakaryocytic‐erythroid progenitors in humans and mice. Blood. 2019;134(18):1547‐1557. 31439541 10.1182/blood.2019002039 PMC6839952

[10] Brissot E , Troadec M‐B , Loréal O , Brissot P . Iron and platelets: a subtle, under‐recognized relationship. Am J Hematol. 2021;96(8):1008‐1016. 33844865 10.1002/ajh.26189

