# Supplementary figures and images for: Inhibition of ferroptosis promotes megakaryocyte differentiation and platelet production
Source: J Cell Mol Med. 2022 May 13;26(12):3582–5. doi: 10.1111/jcmm.17289 (PMC9189328; doi:10.1111/jcmm.17289)

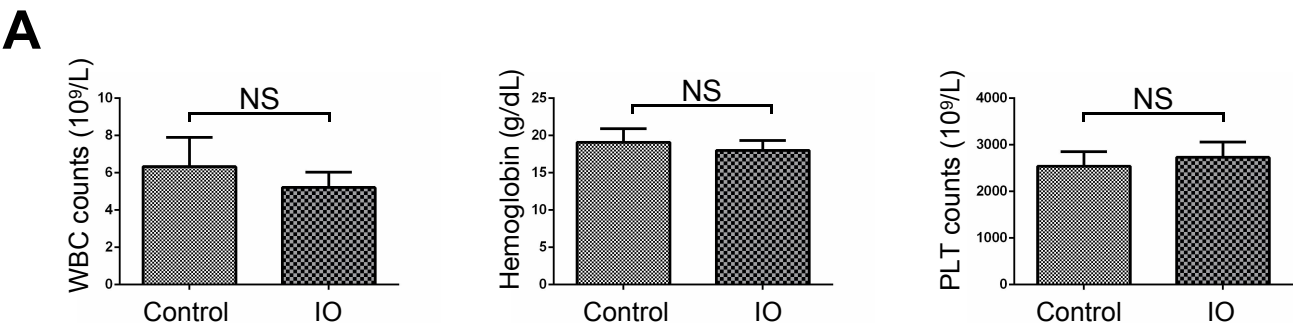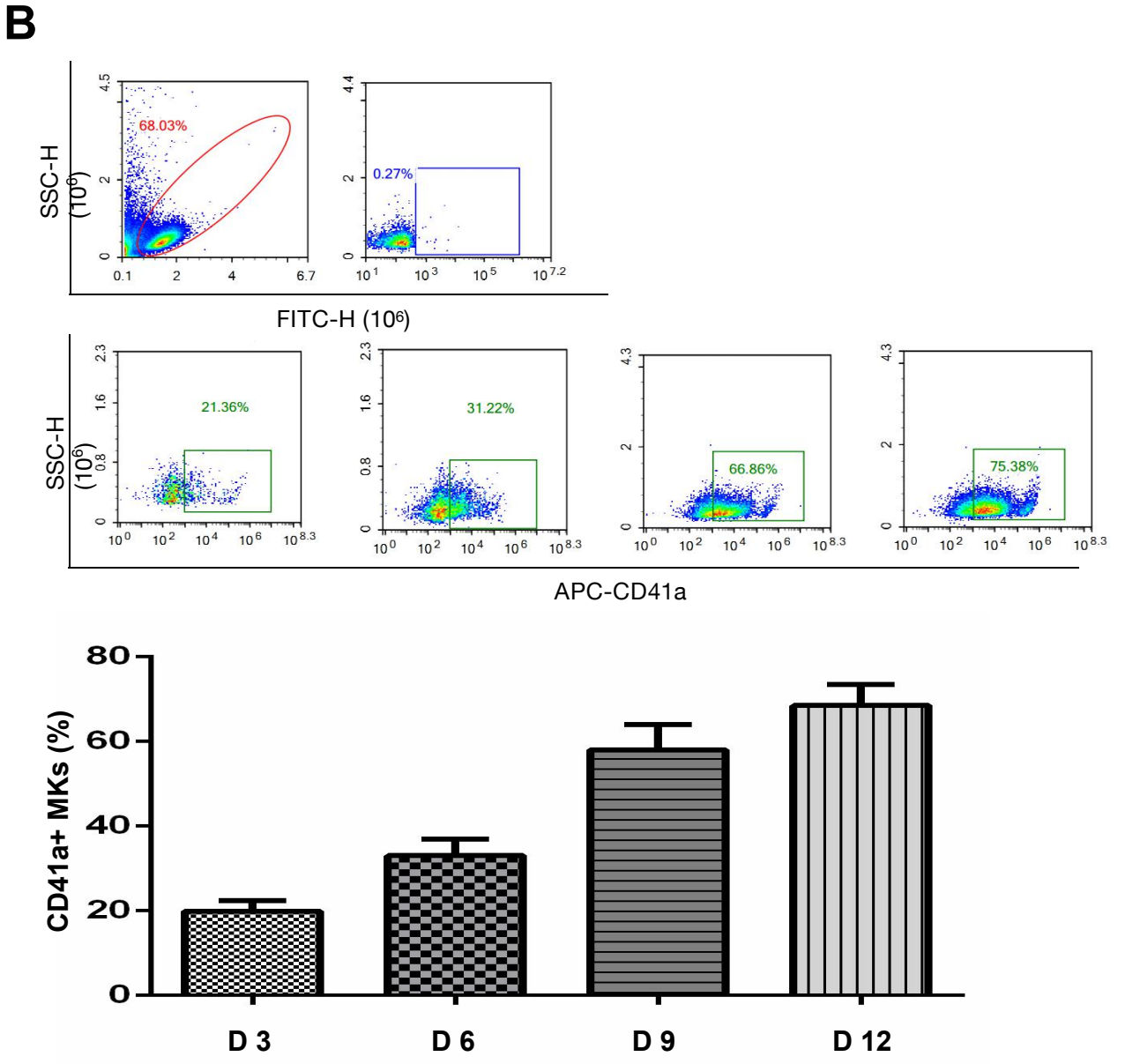

Supplement: Supplementary file 1 — Fig S1 [file JCMM-26-3582-s002.pdf]

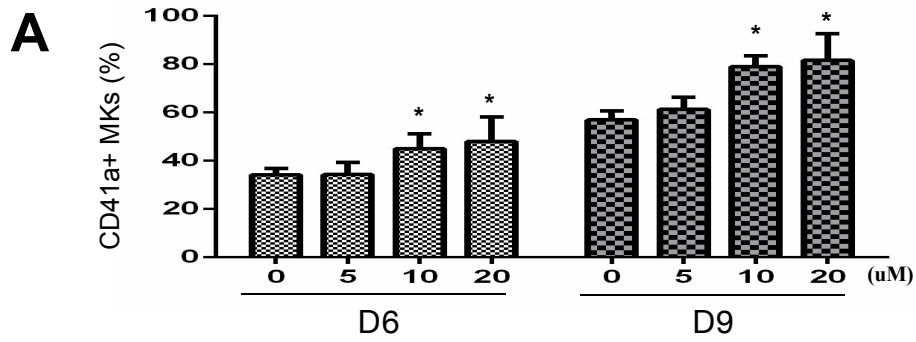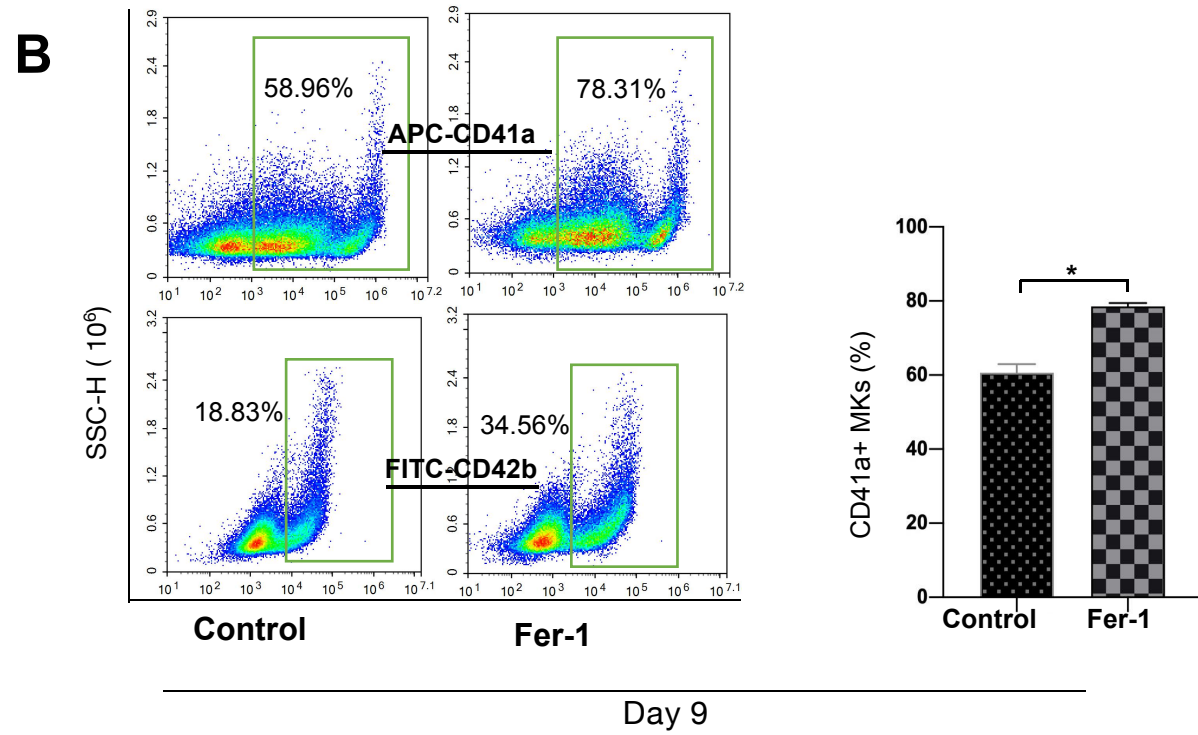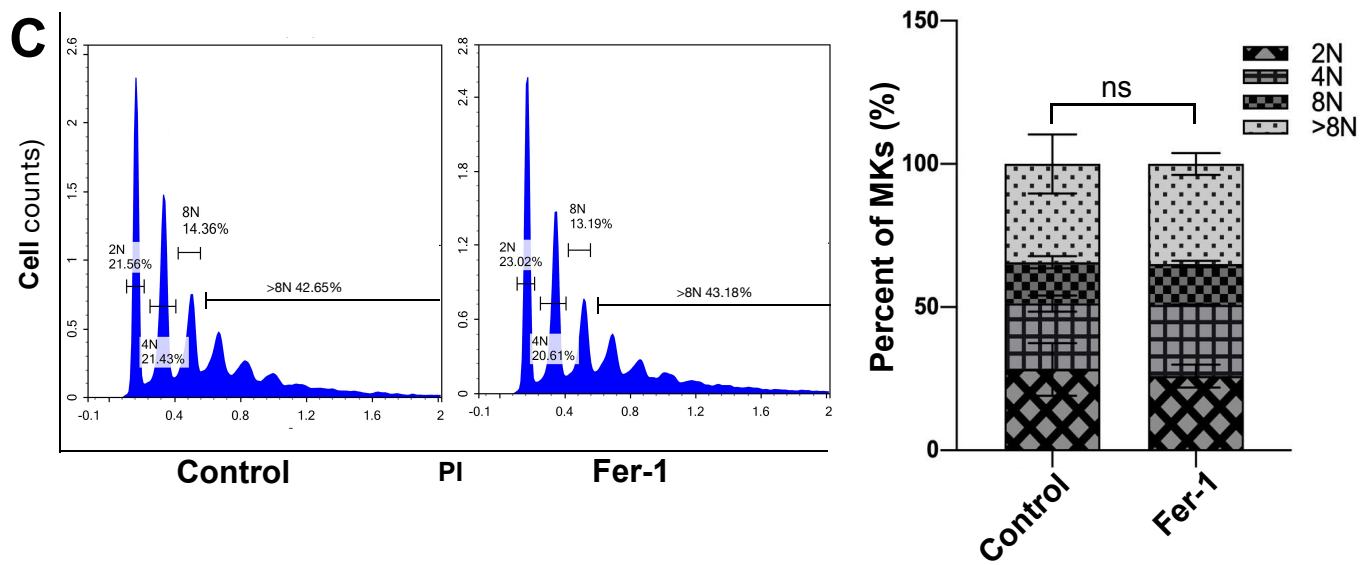

Supplement: Supplementary file 2 — Fig S2 [file JCMM-26-3582-s001.pdf]
