# Supplementary material for: Inhibition of ferroptosis promotes megakaryocyte differentiation and platelet production
Source: J Cell Mol Med. 2022 May 13;26(12):3582–5. doi: 10.1111/jcmm.17289 (PMC9189328; doi:10.1111/jcmm.17289)
Supplement: Supplementary file 3 — Supplementary Material [file JCMM-26-3582-s003.docx]

**Figure S1. A.** Complete blood count in the peripheral blood of control and iron-overloaded mice after 4 wk of injection. WBC: white blood cells, PLT:platelets, NS: not significant. **B.** Flow cytometry analysis for percentage of CD41a+ in megakaryocyte differentiation at different time points.

**Figure S2. A**. Flow cytometry analysis for percentage of CD41a+ during MK differentiation with different Fer-1 concentrations on Day 6 and Day 9. **B.** Flow cytometer analysis for percentage of CD41a+ MKs after Fer-1 treatment at Day 9 of MK differentiation respectively. **C.** Polyploidization analysis of MKs after Fer-1 treatment.
